# “We have been forced to move away from home”: print news coverage of Canadians studying abroad at Caribbean offshore medical schools

**DOI:** 10.1186/s12909-017-1071-4

**Published:** 2017-11-23

**Authors:** Jeffrey Morgan, Valorie A. Crooks, Jeremy Snyder

**Affiliations:** 1 Department of Geography, Simon Fraser University, Burnaby, Canada; 2 Faculty of Health Sciences, Simon Fraser University, Burnaby, Canada

**Keywords:** Offshore medical schools, International medical graduates, Media analysis

## Abstract

**Background:**

Canadian international medical graduates are Canadian-citizens who have graduated from a medical school outside of Canada or the United States. A growing number of Canadians enroll in medical school abroad, including at Caribbean offshore medical schools. Often, Canadians studying medicine abroad attempt to return to Canada for postgraduate residency training and ultimately to practice.

**Methods:**

The authors conducted a qualitative media analysis to discern the dominant themes and ideologies that frame discussion of offshore medical schools, and the Canadian medical students they graduate, in the Canadian print news. We carried out structured searches on Canadian Newsstand Database for print media related to offshore medical schools.

**Results:**

Canadian news articles used two frames to characterize offshore medical schools and the Canadian international medical graduates they train: (1) increased opportunity for medical education for Canadians; and (2) frustration returning to Canada to practice despite domestic physician shortages.

**Conclusion:**

Frames deployed by the Canadian print media to discuss Caribbean offshore medical schools and Canadians studying abroad define two problems: (1) highly qualified Canadians are unable to access medical school in Canada; and (2) some Canadian international medical graduates are unable to return to Canada to practice medicine. Caribbean offshore medical schools are identified as a solution to the first problem while playing a central role in creating the second problem. These frames do not acknowledge that medical school admissions are a primary means to control the make-up of the Canadian physician workforce and they do not address the nature of Canadian physician shortages.

## Background

International medical graduates (IMGs) represent a significant portion of the Canadian physician workforce, accounting for roughly 25% of practicing physicians [1–3]. The Medical Council of Canada defines an IMG as anyone who has graduated from a medical school outside of Canada or the United States (US), including Canadians who study medicine abroad (CSAs) [4]. Most IMGs enter practice in Canada through postgraduate residency training, although most provinces have quotas on the number of placements that are ultimately available to IMGs [5, 6]. As such, obtaining a residency position can be highly competitive, for both CSAs and IMGs [2, 3, 5, 7].

Competition for residency placements in Canada is heightened by the growing number of CSAs in recent decades, which has more than doubled since 2006 and was estimated to be 3500 in 2010 [8]. Canadians study abroad for many reasons, although is primarily associated with the competitiveness of domestic medical school admissions [9]. There are far fewer seats in Canadian medical schools than applicants [10, 11]. For example, in 2014/15 the Association of Faculties of Medicine of Canada reported over 36,000 applications for approximately 2700 places [12]. Canadian medical school enrollment corresponds with attitudes towards physician shortage (or surplus) in Canada, and has fluctuated over time. Current medical school enrollment reflects significant increases between 1997 and 2007, from approximately 1700 seats to 2500 [13]. Nonetheless, the demand for medical education far exceeds domestic supply.

Responding to this demand are medical schools in the United Kingdom, Australia, and Eastern Europe which target and attract Canadian students [8]. Increasingly, the Caribbean region has emerged as a destination for Canadian medical students, building an offshore business model on training international students. Offshore medical schools are private, for-profit enterprises that are purpose-built to provide undergraduate medical education to international students, including from the US and Canada [14–16]. The World Directory lists 49 offshore medical schools currently operating in the Caribbean, of which 22 (48%) opened as of 2007 [17]. The proliferation of this offshore industry has resulted in the Caribbean region having the highest density of medical schools per capita compared to anywhere else in the world [18].

In addition to a serving primarily international student populations, the Caribbean offshore medical school model is unique because students complete two years of basic sciences in the Caribbean followed by two years of clinical sciences abroad, often in the US [15, 16]. Clinical rotations are arranged in the US abroad because the Caribbean region does not have sufficient training capacity [2, 8, 16]. As a result, offshore medical schools routinely purchase clinical placements in the US, dealing directly with hospitals. For example, St. George’s University School of Medicine (SGU), located in the Caribbean country of Grenada, signed an $100 million agreement with New York City’s Health and Hospital Corporation to purchase slots for up to 600 SGU students [16, 19].

Given the fact that offshore medical school graduates must leave the Caribbean practice, and that most CSAs intend on returning to Canada [7, 8], offshore medical schools have been framed as means of addressing physician shortages in graduates’ home countries [14, 20, 21]. The implication is that offshore medical schools can be a source of physicians to lessen shortages left unaddressed by domestic medical schools. That said, the extent to which CSAs (and other IMGs) represent a sustainable solution to addressing physician shortages in Canada is contested, particularly with regard to physician recruitment and retention in underserved rural and remote areas [1, 5, 22]. The nature of physician shortages in Canada, and the role that IMGs and CSAs play in addressing these shortages, are of great debate in the academic and media literature, and is brought forward in our discussion.

Health worker shortages and IMGs receive substantial attention in the media, and effort has been made by researchers to uncover the primary narratives behind the headlines [23–25]. Uncovering media portrayals is important because the media’s coverage of a topic is known to perpetuate certain perspectives and power-dynamics [26], and has influence on public and policy-makers [27]. For example, in the Canadian context, Pylypa [25] showed a notable absence of discussion related to equity impacts of global health worker migration on origin countries discussed in the media when compared to the academic literature [25]. Given the established relationship between offshore medical schools, CSAs, and physician shortages in Canada, in the current article we ask: How are Caribbean offshore medical schools and the CSAs they train framed in the Canadian print media?

## Methods

To our knowledge, this paper constitutes the first media analysis examining offshore medical schools and/or the CSAs they train. Understanding media representions is important because citizen awareness is largely developed through media accounts, which can have direct policy impications [25, 27, 28]. Moreover, news media have shown to provide legitimacy to certain opinions and perspectives [29]. Finally, and importantly in the context of the current under-researched topic, examining publicly available datasets, such as print media, is an empirical first step towards identifying, and in some cases addressing, knowledge gaps. Despite their growing popularity, there is still very little empirical examination of offshore medical schools.

In the current analysis, we take into account how themes are ‘framed’ in and by the media. A framing approach uncovers how some aspects of a phenomenon are focused upon and made salient, while others are ignored [30]. Thus, to understand how a topic is framed is to go beyond considering the information that is reported, but is to also attempt to expose the ideologies and power relations informing how it is presented. As Entman explains: “To frame is to select some aspects of a perceived reality and make them more salient in a communicating text, in such a way as to promote a particular problem definition, causal interpretation, moral evaluation, and/or treatment recommendation for the item described” (p.52) [30]. Framing generally has four functions: (1) *define problems*–determine what an actor is doing with which costs and benefits; (2) *diagnose causes*–identify the forces creating the problem; (3) *offer judgements–*evaluate actors and their impacts; and (4) *offer solutions–*recommend treatments for the problems and predict their outcomes [30].

In October 2015, we conducted structured searches on Canadian Newsstand Database for print media related to Caribbean offshore medical schools. Initially, two Boolean searches were conducted using the search terms: (1) “offshore medical schools” (*n* = 8); (2) “Caribbean” AND “medical schools” (*n* = 405). Consistent with recognized approaches to media analysis, we first reviewed titles of the search results for relevance and duplicates [25]. After this step, we identified 44 unique articles whose content met our inclusion criteria: (1) was published in English; (2) was carried in a Canadian print media source; and (3) dealt with the topic of offshore medical schools and/or returning CSAs. Importantly, included articles did not have to exclusively focus on offshore medical schools and/or their returning CSAs and could instead engage in discussing them in the context of wider issues.

After identifying articles suitable for inclusion, we gained access to their full texts. Print media included in our analysis consisted of: news articles (*n* = 37), editorial and opinion pieces (*n* = 3), and letters to the editor (*n* = 4). By including editorial, opinion pieces, and letters to the editors in our sample, we could capture CSAs perspectives in their own words. Full texts of the 44 articles were imported into NVivo qualitative data management software to organize thematic coding. The thematic codes were broad in order to reflect the themes informing the frames identified in the dataset. The articles were thematically coded by the first author, with input coming from the second author and confirmation from the third author.

## Results

News articles identified by our structured search revealed a wide range of issues related to offshore medical schools and the CSAs they train. Many articles focused on intended students’ decisions to go abroad and their experiences attempting to return to Canada to practice medicine. We found that articles were published consistently (but not in a high volume) since 1983, with a median date of 2006, and a mode of 2013. The most recent article was published in 2015. Table 1 lists the authors, dates, title, and sources of these articles. We identified two frames that print media overwhelmingly used to describe offshore medical schools and the CSAs they train. We include direct quotations from the articles throughout in order to allow the original sources to ‘speak’ to the issues at hand.

**Table 1:** Print news media included in media analysis

Author	Date	Title	Publisher
Oakland, R.	1980	Even out of season, Grenada Carinaval is a joy	The Globe and Mail
Gadd, J.	1983	THE GRENADA INVASION Medical graduates face bleak future	The Globe and Mail
N.A.	1983	Stronghold crushed; general holds out	The Globe and Mail
N.A.	1984	Phony medical papers probed by U.S. states	The Globe and Mail
Rule, S,	1984	Barbs replace bullets Medical school faces siege of different sort	The Globe and Mail
N.A.	1986	Regan in Grenada vows freedom for Nicaragua	The Ottawa Citizen
Gregory, I.P.	1988	Jobless doctor taking case to right panel	The Globe and Mail
Appleby, T.	1989	Grenada – and U.S. self-interest	The Globe and Mail
N.A.	1989	Troops sent to lawless island; Hundreds in fear for lives flee hurricane-ravaged S. Croix	The Ottawa Citizen
Knutzen, E.	1992	Southern Exposure	Toronto Star
Kohanik, E.	1992	Collection of zanies Going to Extremes	The Windsor Star
Kilpatrick, K.	2000	Sunshine with a dash of spice; Scents of nutmeg, cloves and cinnamon add to Grenada’s unique flavour	Toronto Star
Mandal, V.	2001	Lives in Windsor, MD heals in U.S.	The Windsor Star
N.A.	2002	MDs frustrated by lack of acceptance	The Windsor Star
Lu, V.	2002	Ontario to move on doctor shortage; Considers easing restrictions on foreign-trained physicians	Toronto Star
Oakland, R.	2002	Cruising the Canadian Caribbean Islands beckon to pallid Canada; Why not our own island paradise? Survey suggests we could make a deal	Toronto Star
Doughetry, K.	2003	Quebec MD not allowed to practice here	The Montreal Gazette
Lakoff, D.	2004	Footdragging won’t aid health care	The Ottawa Citizen
Wall, M.	2004	It’s not the first time	The Ottawa Citizen
Williamson, D.	2005	Med students forced abroad: Canadians unable to find schooling at home may never return	The Windsor Star
Brook, P.	2006	Forced to study abroad	The Vancouver Sun
Blackwell, T.	2006	Town funds future MD’s schooling in Hungary: Canadian doctor shortage	National Post
Blackwell, T.	2007	Wood-be MDs forced overseas for studies; 1500 Abroad: Estimate	National Post
Ali, N.	2007	Keeping Canada supplied with MDs	National Post
N.A.	2008	Private medical schools are worth studying	The Montreal Gazette
Schwarcz, J.	2009	How do you choose from the brightest and best	The Montreal Gazette
Wray, A.	2010	She’s a role-modelquin	The News
Boesveld, S.	2011	Doctors charged with using date-rape drug on woman, 23	National Post
Harrison, M.	2011	We’re locking doctors out	Telegraph-Journal
French, J.	2011	Private agency hired to recruit doctors	Leader Post
N.A.	2011	Medical school in Caribbean after winning Old Navy contest	The News
Yang, J.	2012	MD claims ‘witch hunt’: Former Scarborough resident accused in U.S. of lying on medical school application and of Medicare fraud vows to fight bid to revoke his license	Toronto Star
Chow, W.	2013	Jane Shin recounts ‘traumatic’ campaign experience	Burnaby News Leader
Evans, G.	2013	Jane Shin should resign	Burnaby Now
Moreau, J.	2013	Newly elected MLA still not commenting	Burnaby Now
Woo, A.	2013	Dix resists urge to return liberal fire	The Globe and Mail
McDiarmid, J.	2013	Student survives brutal stabbing in St. Kitts: Samar Haroun’s horror began with a knife-weilding man in her apartment closet	Toronto Star
Oosenburg, M.	2013	Tomorrow’s physicians	The Gazette
Sherlock, T.	2013	World’s top schools courting Canadian students	The Vancouver Sun
Barer, M. & Evans, R.	2013	Canada will soon face a glut of new physicians	Star – Phoenix
Barer, M. & Evans, R.	2013	Canada is heading toward an over-supply of MDs	The Province
Olsen, T.	2014	Winner of $100,000 prize fulfills promise	The News
Cummings, M.	2015	Murder suspect released from custody in Saba	Edmonton Journal
Pruden, J.G.	2015	Canadian held in death of U.S. medical student; Police on Caribbean island say probe yielded ‘incriminating evidence’	Calgary Herald

### Increased opportunity for medical education due to unreasonable competition in Canada

Many of the articles framed students’ decisions to attend Caribbean offshore medical schools around the competitiveness of Canadian medical school admissions. Specifically, many CSAs were perceived as having been “pushed abroad” by the competitiveness of domestic universities. Similarly, Canadian medical schools were seen as unable (or unwilling) meet the growing demand for undergraduate medical education. As a result, Caribbean offshore medical schools were portrayed as increasing opportunities to study medicine. Several articles pointed out that some students who met all admissions criteria, and were thus seemingly qualified, were nonetheless rejected from domestic medical programs:

*At present, public medical schools in Canada have neither the money nor the room to accept all qualified candidates… [and can] admit no more than one-quarter of the students who apply* [31].

Offshore medical schools were thus framed offering spaces for *qualified* Canadians who were denied entry into domestic programs, in the face of growing demand and intense competition.

Some media sources used a narrative approach to highlight the factors that pushed Canadians to study abroad. Narratives typically revealed glowing personal profiles of students who were rejected from Canadian medical schools. For example:

*[P]erfectly bilingual, [the student] had an A+ average coming out of her B.Sc…where she spent her free time shadowing a family physician and volunteering at camps and after-school programs for underprivileged kids… ‘It [admission to a Canadian medical school] seemed totally unattainable,’ [the student] says* [32].

*[S]he [the student] had applied to numerous schools in Canada but wasn’t accepted due to limited spots, despite her high scores on her Medical College Admission Test and volunteer work at BC Children’s Hospital* [33].

These excerpts frame Canadian students’ attendance at offshore medical schools as a logical choice in the face of unrelenting competition at domestic schools, even for those who scored high on admission tests, achieved excellent grades, and had undertaken related volunteer work. This constrained decision-making was exemplified by the frustrated remarks of one student: “*I mean, what else could I have done?”* [32].

### Frustration returning to Canada in the face of physician shortages

The second frame that Canadian print media used in reference to offshore medical schools and the CSAs they train pertained to graduates’ frustration in attempting to return to Canada for postgraduate residency training, and ultimately to practice. This was attributed to limited postgraduate residency opportunities in Canada, which are closely matched to the number of domestic medical school seats [5, 7]. Many articles paralleled difficulties returning to Canada against claims of domestic physician shortages. These articles suggested that CSAs who have graduated from offshore medical schools and other international institutions are ideally placed to address shortages, but that their opportunities to do so are limited by practical barriers. As expressed in these opinion and editorial pieces written by an offshore medical school graduates:

*We have been forced to move away from home, due to the limited spaces available in medical schools in Canada. Ontario [a Canadian province] has long been complaining about its physician shortage, but why not give us internationally trained graduates a chance to contribute to our ailing health care system?* [34]

*On completion of our medical training, many of us are very interested in returning to our home and native land, but we are being locked out by the bureaucracy…It is becoming apparent that the federal and provincial governments, through combined foot-dragging, are not doing enough to help Canadians build a stronger health-care system. If they were truly serious about the problem, they would put their money where there mouths are, instead of in their pockets* [35]*.*

By positioning difficulty accessing residency placements in Canada alongside narratives of physician shortages, many articles framed this situation as a lost opportunity for the Canadian healthcare system.

Bureaucratic barriers were commonly reported as perceived obstacles to CSAs practicing in Canada upon graduation:

*I met a large number of outstanding Canadian students at St. Georges [in Grenada] who would love to come back and practice in this country but find it difficult because of regulatory hurdles placed in their path. In light of our current shortage of physicians this is most unfortunate* [36].

*Students…want to fill that physician gap, but our government-controlled medical schools have no room for them* [37].

*Canadian schools are not producing enough doctors at present. Off-shore schools…are producing high-quality physicians at low costs to the Canadian system. But, bureaucracy is keeping Canada from tapping this source to its full potential* [38].

This framing implicitly positions offshore medical schools as quality medical training institutions and their “*outstanding*” graduates as ready and able to practice in Canada. Such positioning heightens frustration around reported bureaucratic barriers that prevent CSAs from helping to reduce Canada’s perceived physician shortage.

Policy decisions to give CSAs equal, rather than preferred, access to Canadian residency placements as other IMGs upon graduation and return to Canada were critiqued by some media sources. These policies were often presented as unfair:

*Even though they are citizens of this country [Canada], they would be classified as international medical graduates and would have to get in line with foreign-trained doctors to re-enter the Canadian system* [37].

*Getting a chance to practise back home can be the biggest challenge of the ex-patriots’ global educations. They are treated like any other international medical graduates who apply for the residency training positions required before they can practise here…the majority still end up in the United States* [39].

As these articles note, many CSAs who trained at offshore medical schools ultimately end up practicing in the US despite interest in working in Canada. Articles consistently framed excellent Canadian students as being pushed abroad to offshore medical schools due to the limited number of seats available at Canadian universities and then, upon graduation, being pushed to practice in the US because of bureaucratic decisions to treat them equal to all other IMGs in Canada.

## Discussion

This analysis has identified two ways that the Canadian print media frame offshore medical schools and the CSAs they train. We contend that our focus on framing is useful as it has drawn attention to how communicated texts reveal dominant meanings in the context of this particular dialogue [29]. Following Entman’s framing paradigm [30], framing has four functions: defining problems, diagnosing causes, making judgements, and suggesting solutions. In this section we first consider what is learned about the four functions of the two frames uncovered by this analysis, also summarized in Table 1. As framing accounts for how some aspects of a phenomenon are made salient while others are ignored, we also consider the ideologies and power dynamics that inform these dialogues and look to the relevant literature to identify important points left unaddressed by the Canadian print media (Table 2).

**Table 2:** Summary of two frames presented in Canadian print media

	Increased opportunity for medical education due to unreasonable competition in Canada	Frustration returning to Canada in the face of domestic physician shortages
Define Problem	Canadian medical schools have unreasonable admission standards for medical school	Canadian medical schools and provinces have made it too difficult to return to Canada
Diagnose Causes	There are too few medical schools in Canada	There are too few residency placements
Evaluate Effects	Students are driven abroad to find alternative medical schools	Students are forced to practice in the US or unable to practice medicine
Suggest Remedies	Canada should accept more medical students; Offshore medical education model	Canada should make it easier for graduates to return to Canada

### Engaging the four functions of framing

Dominant frames presented by the Canadian print media to discuss offshore medical schools and the CSAs they train identified two problems: (1) qualified Canadians are unable to access domestic medical schools; and (2) CSAs are unable to return to Canada to practice medicine despite a perceived shortage. Frames presented Canadian medical schools and provincial healthcare administrators as principal casual agents, suggesting that both problems are the result of their (poor) decision-making. These same frames positioned offshore medical schools as a solution to the first problem. An inadequate number of domestic medical school seats and residency placements were portrayed as primary causes, burdened by bureaucracy. As a result, students were pushed abroad–both to study, and ultimately practice, medicine, which was understood as lost potential.

References made to bureaucracy by the reviewed media sources typically served as a critique of Canadian medical schools and provincial healthcare administrators. We conceptualize bureaucracy as an administrative structure that is rational, rule-bound, hierarchical, and typical of those found in governmental and academic institutions. For example, the perceived inadequate supply of medical training capacity was understood in terms of insufficient funding, which is a bureaucratic decision. As suggested in the findings, Canadian medical schools were seen as having “*neither the money nor the room to accept all qualified candidates”* [31]. This positioning implicitly suggests Canadian medical schools *should* accept all qualified candidates, but do not because of funding and administrative shortfalls. With regard to the difficulty faced by CSAs in returning to Canada to practice, bureaucracy was framed as red-tape. As expressed in the findings: “*[CSAs] find it difficult because of regulatory hurdles placed in their path”* [36]*.* In this way, bureaucracy was framed something to be overcome by CSAs.

Both frames understood the adverse effects of bureaucracy as lost potential for the Canadian healthcare system. First, lost potential was conceptualized as Canadian medical schools being unable to accept all qualified applicants. This has been echoed elsewhere, such as in a qualitative study led by Wong & Lohfeld (2008) that found that IMGs in Canada, including CSAs [3], routinely report difficulty accessing residency training, despite perceived physician shortages. Their participants characterized bureaucracy as contributing to the problem, including the “ambiguous selection criteria and lack of feedback” (p.55) [3]. As a result, students who have the potential to make excellent physicians were denied the opportunity to train domestically and/or forced to go abroad. Second, lost potential was articulated as ‘brain drain,’ or CSAs being forced to practice medicine elsewhere, usually in the US.

### Underlying ideologies of the frames

From this analysis, we contend that both these frames signal towards ideologies of neoliberalism, and power dynamics that reveal privileging of citizenship and entitlement. Neoliberalism is an established political ideology that, among other things, advocates for the retrenchment of government regulation and bureaucracy, encouraging free-markets and privatization [40]. With regard to the provision of health services, neoliberal ideologies increasingly cast patients as ‘clients’ and ‘customers’ [41] and advocate for the privatization of health services, including post-secondary institutions [42]. Offshore medical schools and the CSAs they train can thus be viewed as products of neoliberalism, while also benefitting from global neoliberalism in many ways–such as via trade liberalization policies that facilitate mobility between countries as well as offshoring industries. Neoliberal ideologies also informed critiques raised by media coverage, such as that medical school seats in Canada are allocated by perceived need rather than by letting the ‘market’ decided the supply based on demand. We believe that understanding dominant media frames as being informed by neoliberal ideology helps to contextualizes these narratives while situating this industry alongside other forms of international medical mobility, such as medical tourism, that are similarly thought to be perpetuated by neoliberal policies and practices [41].

Frames brought forward in the media analysis also reveal instances of privilege and entitlement. For example, most articles portrayed CSAs as not only feeling entitled to access medical education, but also to holding residency placements in Canada. This entitlement is reflected in statements such as *“we were forced to move away from home”* [34] and *“what else could I have done?”* [32]*.* It is also clear that frames project a privileging of citizenship, as made clear in repeatedly expressed frustration that CSAs are treated the same as non-Canadian IMGs. For example, as one article noted *“Even though they [CSAs] are citizens of this country, they would be classified as international medical graduates and would have to get in line with foreign-trained doctors”* [37]. That said, some have questioned whether granting CSAs greater access to postgraduate training would be ethical or legally justifiable, as this would provide preferential treatment on the basis of country of birth, which could violate the Charter of Rights and Freedoms [43]. What we cannot know from the current analysis is if notions of privilege and entitlement are common among Canadians studying at offshore medical schools and returning CSAs, or if the voices represented in the media are the most privileged and/or outspoken and thus not particularly reflective of this group as a whole.

### Critical points unaddressed by the frames

Because a framing approach to analysis requires consideration of what is ignored in a text, here we consider what was left unaddressed by the Canadian print media. First, with regard to competition in the Canadian medical school applicant pool, what is left unaddressed is that admission protocols and class sizes are not merely a reflection of available resources. In fact, the number of medical school seats have increased substantially in the last decade [5, 9]. Rather, in addition to reflecting provincial budgets and available resources, medical school admissions are a primary means to control the size and diversity of the Canadian physician workforce [5], in addition to a way of balancing. In this way, bureaucracy is a deliberate mechanism for health human resource planning, rather than simply a barrier to be overcome as suggested by several of the reviewed media articles.

With regard to the difficulty faced by many CSAs returning to Canada for residency and ultimately practice despite reports of physician shortages, controlling the number of post-graduate positions is an instrumental way to control the make-up of the physician workforce [44]. Further, this framing also does not address the nature of physician shortages in Canada, which are better characterized as rural-urban physician mal-distributions [45]. There is evidence to suggest that admitting IMGs for residencies and ultimately practice is not an effective way to address physician mal-distribution in Canada as these physicians often migrate to urban centers and not to underserviced areas of the country [46–49]. Instead, it is believed that recruiting more students from underserved areas will better aid in addressing mal-distribution, as they are more likely to return to serve these same populations [46, 50].

### Future research directions

This analysis represents a novel approach to understanding Canadian dialogue surrounding offshore medical schools and the CSAs they train. Future research questions related to the current analysis include: what frames do other stakeholder groups use when discussing offshore medical schools; and what insights can current and past Canadian offshore medical school students offer into understanding the two frames identified in this analysis? There are also broader questions to be asked, such as: what awareness do prospective offshore medical school students have of their employment opportunities upon graduation; and what role(s) should offshore medical school graduates play in addressing health worker shortages? Answering such questions will require a diversity of research designs and are best addressed through interdisciplinary approaches and engagement with diverse stakeholder groups.

## Conclusions

Offshore medical schools are for-profit, private enterprises located in the Caribbean that provide undergraduate medical education for mostly international students. Many of these students seek to practice medicine in their home countries, including the US and Canada. This research has shown two ways that public discussion of offshore medical schools is framed in the Canadian print media. First, it is suggested there is unreasonable competition for Canadian medical schools, and some qualified students seeking admission are pushed abroad to offshore medical schools. Second, Canadian graduates of offshore medical schools face difficulty returning to Canada, even in the face of perceived physician shortages. Both frames identify Canadian medical schools and provincial healthcare administrators as causal agents, burdened by bureaucracy. Both frames understand these problems to have the adverse effect of lost health worker potential for the Canadian healthcare system. Meanwhile, as pointed out in the discussion, we contend that domestic medical school seats and residency opportunities are important tools used to control the Canadian physician workforce, which was left unaddressed by these framings. Given the increasing popularity of Canadians perusing education in Caribbean offshore medical schools, we call for more research into this area, including that which aids in further unpacking the two frames identified in the present analysis.

## Abbreviations

CSA: Canadians studying medicine abroad
IMG: International medical graduate
SGU: St. George’s University School of Medicine
US: United States
